# Cortisol Responses to a Laboratory Challenge: The Moderating Role of Cognitive Performance

**DOI:** 10.1093/geroni/igaa057.1166

**Published:** 2020-12-16

**Authors:** Elise Grimm, Stefan Agrigoroaei

**Affiliations:** 1 Universite Catholique de Louvain, Louvain-La-Neuve, Belgium; 2 UCLouvain, Belgium, Louvain-La-Neuve, Belgium

## Abstract

Recent theoretical and empirical studies have considered higher cognitive performance as a protective factor with respect to reactivity, recovery and habituation to acute stressors. The goal of our study was to examine the individual role of inhibition, working memory, processing speed, reasoning, and category fluency in the regulation of the cortisol response to a laboratory challenge. Younger, middle-aged, and older participants (N =109, aged 22-84, M=55.90, SD=16.35) were invited to a laboratory session comprising a driving simulation and a set of cognitive tasks. At least one week in advance, baseline cognitive performance was measured using the Brief Test of Adult Cognition by Telephone (BTACT). Throughout the lab session, five saliva samples were taken, which allowed for the computation of a global measure of cortisol release (area under the curve (AUC)). Cortisol AUC was regressed on the individual BTACT cognitive tests, while controlling for age, sex, education, body mass index, physical activity, and time since awakening. The results revealed that inhibition and working memory significantly accounted for the cortisol response. These associations remained significant when other factors such as smoking, caffeine consumption, and medication use were included as covariates. The contributions of reasoning and speed of processing approached significance. Our findings contribute to the emerging evidence that cognitive functioning modulates stress responses to acute stressors. The findings are discussed in the context of cognitive interventions with transfers and implications for stress processes and healthy aging.

